# A Tabular History and Analysis

**Published:** 1871-01

**Authors:** 


A Tabular History and Analysis of all the Undoubted Cases of Typhoid and Typhus Fevers, Presented at the Boston City Hospital, from June 1, 1864 to June 1, 1869. By J. Baxton Upham, A.M., M.D., Late Visiting-Physician to the Hospital. Reprinted from the First Quinquennial Volume of the Hospital Reports. Boston: 1870.

This is an elegantly printed monograph of 93 pages, into which are condensed all the important points concerning the history and treatment of 152 cases of typhoid, and 48 cases of typhus fevers.

The author is one of the most industrious and careful observers in the profession, and the present report is a valuable addition to the literature of continued fevers. Being mostly tabular and statistical, it is not easy to give a resume, but advise our readers to peruse the report itself.

